# Investigation of the halo-artifact in ^68^Ga-PSMA-11-PET/MRI

**DOI:** 10.1371/journal.pone.0183329

**Published:** 2017-08-17

**Authors:** Thorsten Heußer, Philipp Mann, Christopher M. Rank, Martin Schäfer, Antonia Dimitrakopoulou-Strauss, Heinz-Peter Schlemmer, Boris A. Hadaschik, Klaus Kopka, Peter Bachert, Marc Kachelrieß, Martin T. Freitag

**Affiliations:** 1 Medical Physics in Radiology, German Cancer Research Center (DKFZ), Heidelberg, Germany; 2 Applied Medical Radiation Physics, German Cancer Research Center (DKFZ), Heidelberg, Germany; 3 Divison of Radiopharmaceutical Chemistry, German Cancer Research Center (DKFZ), Heidelberg, Germany; 4 Clinical Cooperation Unit Nuclear Medicine, German Cancer Research Center (DKFZ), Heidelberg, Germany; 5 Department of Radiology, German Cancer Research Center (DKFZ), Heidelberg, Germany; 6 Department of Urology, University Hospital of Essen, Essen, Germany; University of South Australia, AUSTRALIA

## Abstract

**Objectives:**

Combined positron emission tomography (PET) and magnetic resonance imaging (MRI) targeting the prostate-specific membrane antigen (PSMA) with a ^68^Ga-labelled PSMA-analog (^68^Ga-PSMA-11) is discussed as a promising diagnostic method for patients with suspicion or history of prostate cancer. One potential drawback of this method are severe photopenic (halo-) artifacts surrounding the bladder and the kidneys in the scatter-corrected PET images, which have been reported to occur frequently in clinical practice. The goal of this work was to investigate the occurrence and impact of these artifacts and, secondly, to evaluate variants of the standard scatter correction method with regard to halo-artifact suppression.

**Methods:**

Experiments using a dedicated pelvis phantom were conducted to investigate whether the halo-artifact is modality-, tracer-, and/or concentration-dependent. Furthermore, 31 patients with history of prostate cancer were selected from an ongoing ^68^Ga-PSMA-11-PET/MRI study. For each patient, PET raw data were reconstructed employing six different variants of PET scatter correction: absolute scatter scaling, relative scatter scaling, and relative scatter scaling combined with prompt gamma correction, each of which was combined with a maximum scatter fraction (MaxSF) of MaxSF = 75% or MaxSF = 40%. Evaluation of the reconstructed images with regard to halo-artifact suppression was performed both quantitatively using statistical analysis and qualitatively by two independent readers.

**Results:**

The phantom experiments did not reveal any modality-dependency (PET/MRI vs. PET/CT) or tracer-dependency (^68^Ga vs. ^18^F-FDG). Patient- and phantom-based data indicated that halo-artifacts derive from high organ-to-background activity ratios (OBR) between bladder/kidneys and surrounding soft tissue, with a positive correlation between OBR and halo size. Comparing different variants of scatter correction, reducing the maximum scatter fraction from the default value MaxSF = 75% to MaxSF = 40% was found to efficiently suppress halo-artifacts in both phantom and patient data. In 1 of 31 patients, reducing the maximum scatter fraction provided new PET-based information changing the patient’s diagnosis.

**Conclusion:**

Halo-artifacts are particularly observed for ^68^Ga-PSMA-11-PET/MRI due to 1) the biodistribution of the PSMA-11-tracer resulting in large OBRs for bladder and kidneys and 2) inaccurate scatter correction methods currently used in clinical routine, which tend to overestimate the scatter contribution. If not compensated for, ^68^Ga-PSMA-11 uptake pathologies may be masked by halo-artifacts leading to false-negative diagnoses. Reducing the maximum scatter fraction was found to efficiently suppress halo-artifacts.

## Introduction

Since the prostate-specific membrane antigen (PSMA) shows substantially increased expression in primary and recurrent prostate cancer cells [*1*,*2*], positron emission tomography (PET) targeting PSMA has been proposed for sensitive imaging of recurrent prostate cancer [*3*]. Especially the introduction of Glu-NH-CO-NH-Lys-[^68^Gallium-(HBED-CC)] (^68^Ga-PSMA-11) [*4*,*5*] is considered to have substantially improved detectability and staging of prostate cancer in both PET/CT [*5*–*10*] and, more recently, PET/MRI [*11*–*14*]. Particularly, ^68^Ga-PSMA-11-PET/MRI is very promising as both the excellent diagnostic performance of ^68^Ga-PSMA-11-PET for lymph node metastases [*9*,*13*] and the high spatial resolution and soft tissue contrast of MRI, beneficial for imaging the pelvis/abdomen, are combined while minimizing radiation exposure compared to PET/CT.

PSMA is scarcely expressed in abdominal fat and soft tissue while a large proportion of ^68^Ga-PSMA-11 is excreted by the urine and collected within the urinary system [*5*], resulting in extreme activity concentration differences between bladder/kidneys and surrounding background. Thus, large organ-to-background activity ratios (OBRs) sometimes result in photopenic artifacts surrounding the kidneys and the bladder in both PET/CT [*8*,*15*,*16*] and, in particular, PET/MRI [*11*,*13*,*17*]. These so-called halo-artifacts pose one potential drawback of the clinical use of ^68^Ga-PSMA-11-PET/MRI for prostate cancer detection and staging. Both primary prostate cancer and local recurrences after radical prostatectomy are typically located very close to the bladder and any photopenic artifact surrounding the bladder could potentially mask findings, falsify uptake values and thus influence the patients’ diagnosis. Additionally, retroperitoneally between the kidneys, the halo may, potentially, mask metastases and thus impair tumor detectability and staging.

**Fig 1:**
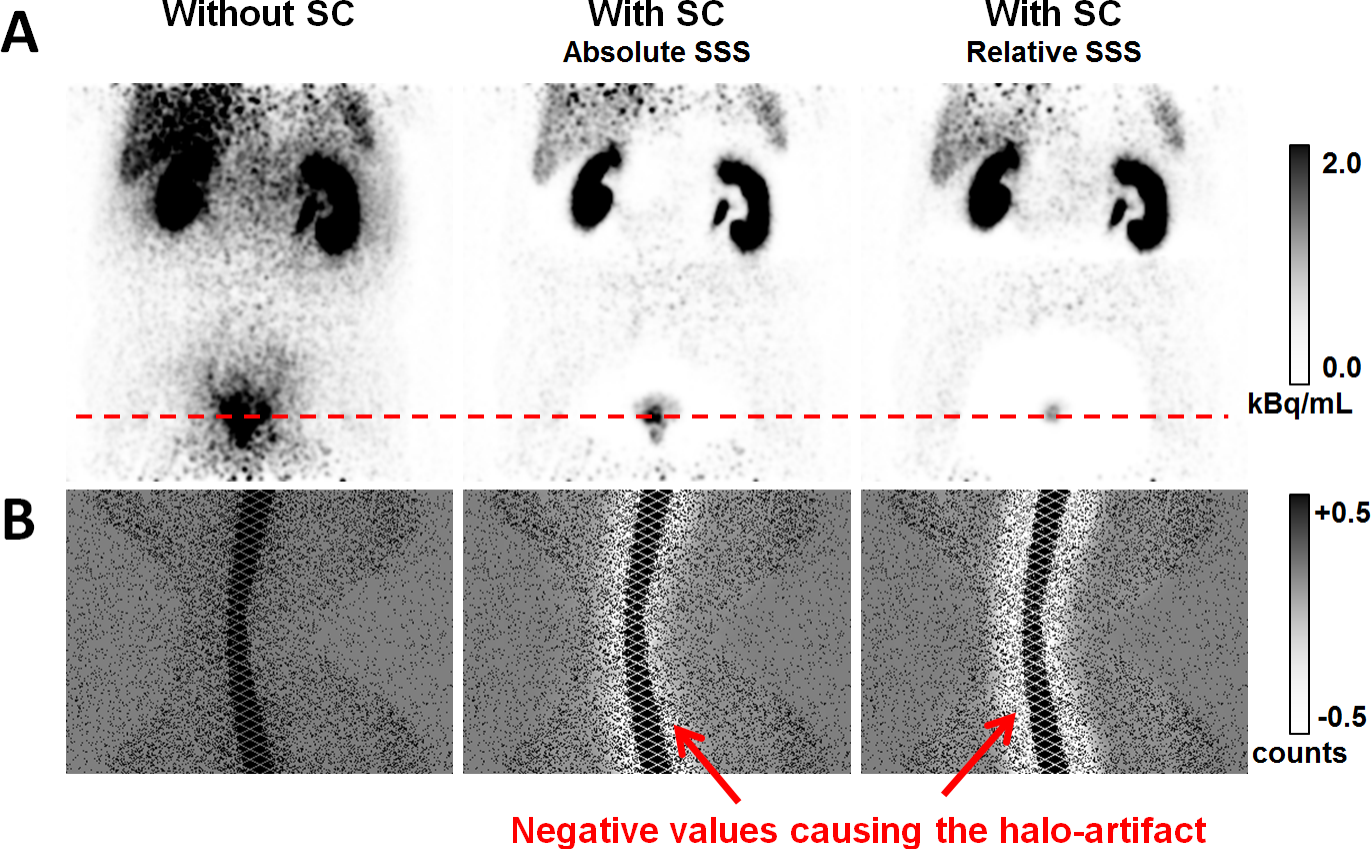
Example for halo-artifacts surrounding the urinary bladder and the kidneys. (A) PET images in coronal slice orientation for a 59 years old patient with and without scatter correction (SC). Administered activity was 144 MBq of ^68^Ga-PSMA-11 and the patient was scanned with the mMR 104 min p.i. Absolute SSS resulted in a strongly reduced halo-artifact around the bladder compared to relative SSS. (B) Sinograms corresponding to the direct plane indicated by the dashed red line. Shown are the prompts after subtraction of the estimated scatter. Note the grayscale windowing, which is chosen such that white color indicates negative values.

There is evidence that halo-artifacts in ^68^Ga-PSMA-PET/MRI are caused by scatter correction [*11*,*17*,*18*]. The most widely used scatter correction technique in clinical PET imaging is based on the single scatter simulation (SSS) algorithm [*19*,*20*], which analytically estimates the scatter contribution from a coarse initial activity image by employing a simplified physical model. SSS explicitly models only single scatter events while multiple scatter and other physical aspects like scatter from activity located outside the field of view (FOV) are neglected. Due to the simplifications and the potentially inaccurate initial activity estimate, SSS can only estimate the shape of the scatter contribution but not its magnitude [*19*]. The resulting shift of the estimated scatter contribution with respect to the measured emission data requires additional scaling. There are two different methods to perform scatter scaling which are currently available in clinical practice. Relative scaling scales the estimated scatter obtained by SSS to fit to the scatter tails of the measured PET emission data, i.e., to the measured events outside the patient outline, which are assumed to stem from scatter events only. Absolute scaling, on the other hand, also estimates the distribution of unscattered events based on the initial activity estimate and employing a forward model. This allows to relate the SSS-based scatter estimate to the estimated unscattered component, which further allows for an intrinsic scaling of the estimated scatter, without the need to access the emission data [*20*]. In principle, relative SSS and absolute SSS differ only in the way the SSS scatter estimate is scaled, i.e., only the magnitude of the final scatter, not its shape is different. However, since the scatter estimate is usually refined iteratively, differences in the initial magnitude may also alter the scatter shape throughout the iterative process, such that both magnitude and shape obtained with relative and absolute SSS differ in general,

While relative scaling can in principle also compensate for multiple scatter and for scatter contributions from activity located outside the FOV, this is not the case for absolute scaling. This may be one reason why relative scaling is the default in clinical PET imaging. For highly specific PET tracers, such as ^68^Ga-PSMA-11 for example, our experience from daily clinical practice indicates that images based on absolute SSS are, however, less prone to halo-artifacts as compared to relative SSS (Fig 1A).

Fig 1B presents 2D sinograms corresponding to the direct plane indicated by the dashed red line. The sinograms show the measured prompt events (prior to gap filling) after subtraction of the scatter estimates. Without scatter correction, the scatter estimate is zero, and the respective sinogram shows the unmodified prompts. In case of SSS-based scatter correction, the presented sinograms contain negative values for a large amount of lines of response (LORs) crossing the region surrounding the bladder. These negative values indicate that the estimated scatter is larger than the measured prompt events for the corresponding LORs. From a physical point of view, this does not make sense, since the prompts are the sum of the measured true, scattered, and random events. If the scatter estimate exceeds the measured prompts, the reconstruction algorithm, usually ordered subset expectation maximization (OSEM) in clinical PET imaging, enforces zero-valued voxels in the affected region. If there was no non-negativity constraint as with OSEM, the voxels surrounding the bladder would even be assigned negative values, as it is the case with the filtered backprojection algorithm implemented by the vendor. The example presented in Fig 1 impressively demonstrates that the current implementation of SSS may result in scatter overestimation, which is the cause of halo-artifacts frequently observed in ^68^Ga-PSMA-PET/MRI.

Furthermore, in ^68^Ga decay, prompt or cascade gammas emitted simultaneously with positron emission occur with a branching ratio of around 1.2% [*21*]. The gammas have an energy of 1077 keV and are thus way above the energy window of all clinical PET scanners (e.g., 430 to 610 keV for the Siemens Biograph mMR). If, however, the gammas are scattered prior to being detected, their energy may fall within the energy window. In such a case, coincidences between prompt gammas and annihilation photons cannot be distinguished from true coincidences and degrade the image quality if not compensated for [*22*]. Prompt gamma correction (PGC) has been shown to strongly improve PET quantification using ^76^Br, ^82^Rb, ^86^Y, and other isotopes having a much higher prompt gamma branching fraction than ^68^Ga [*23*–*25*]. More recently, PGC has also been shown to reduce halo-artifacts around the kidneys in PET/CT [*15*]. Ignoring prompt gammas may result in scatter overestimation when applying relative SSS [*23*,*25*]. However, PGC is currently not employed in routine clinical ^68^Ga-PET imaging. In a recently published study, Noto et al. showed that PGC only has a limited effect on the appearance of halo-artifacts in ^68^Ga-PSMA-PET/MRI [*18*].

To the best of our knowledge, there is no publication providing a methodological investigation of the halo-artifact in ^68^Ga-PSMA-11-PET/MRI. However, there are two recent publications demonstrating a positive impact of arm truncation correction on the appearance of halo-artifacts [*18*,*26*]. In the present study, we performed phantom experiments using a dedicated pelvis phantom investigating modality-, tracer-, and OBR-dependency of the halo-artifact. Moreover, we retrospectively collected data from ^68^Ga-PSMA-11-PET/MRI patient scans revealing halo-artifacts, characterized their size in correlation with the OBR, and evaluated several variants of SSS. We performed quantitative and visual assessments and evaluated which of the SSS variants achieves best image quality in terms of halo-artifact suppression.

## Materials and methods

Phantom and patient measurements were conducted with a clinical non time-of-flight (TOF) PET/MRI system (3 Tesla Biograph mMR, Siemens Healthineers, Erlangen, Germany) [*27*]. Comparative phantom measurements were additionally conducted on a clinical TOF PET/CT system (Biograph mCT, Siemens Healthineers, Erlangen, Germany) [*28*].

### Phantom measurements

Measurements were performed using a dedicated pelvis phantom [*29*] consisting of an 80 mL bladder insert enclosed by an 11 L polymethyl-methacrylate box mimicking the soft tissue background. The maximum outer dimensions of the phantom are about 40 cm within the transaxial plane and about 20 cm in axial direction, such that it can be entirely covered by a single bed position both with the mMR and the mCT. Both bladder and background were filled with deionized water and 0.9% NaCl for all measurements. For PET/MRI, we used a co-registered PET/CT-based attenuation map as basis for attenuation and scatter correction to avoid artifacts caused by the MR-based attenuation map not considering the phantom box. For each individual measurement described in the following, the phantom was placed on the patient bed such that the bladder insert was located in the center of the PET FOV both within the transaxial plane and along the axial direction. Data were acquired for a single bed position. If not state otherwise, 10^7^ counts (prompts after randoms correction).were acquired in sinogram mode.

#### PET/MRI vs. PET/CT

Experiments were conducted to qualitatively investigate halo occurrence and size in PET/MRI in comparison to PET/CT. Therefore, 30.0 MBq of ^68^Ga were administered to the bladder and 5.3 MBq to the background. The phantom was scanned successively in PET/CT and PET/MRI, with data being acquired for approximately 1 min in both cases.

#### ^68^Ga vs. ^18^F-FDG

In contrast to ^68^Ga, no prompt gammas are present in the decay of ^18^F. Therefore, to investigate the influence of prompt gammas on the halo-artifact, the PET/MRI vs. PET/CT experiment was repeated, replacing ^68^Ga with ^18^F-FDG. 30.0 MBq of ^18^F-FDG were administered to the bladder and 5.3 MBq to the background. The phantom was scanned successively in PET/CT and PET/MRI, with data being acquired for approximately 1 min in both cases.

#### Dual tracer experiment

Annihilation photons originating from different PET tracers are indistinguishable and there is no physiological uptake to be considered during phantom measurements. Therefore, if different tracers are administered to bladder and background, changes in relative activity values are only affected by the different half-lives of the radioisotopes. Thus, the dual-tracer approach is an elegant method to obtain time-dependent OBRs under controlled conditions. We administered 33 MBq of ^68^Ga to the background and 23 MBq of ^18^F-FDG to the bladder. Due to the longer half-life of ^18^F (T_1/2_ = 110 min) compared to ^68^Ga (T_1/2_ = 68 min), the OBR increases over time with an expected doubling every 178 min. The measurements were performed on the mMR and PET raw data were acquired 15, 75, 135, and 195 min post-injection (p.i.), with data acquisition times of approximately 0.8, 1.5, 2.5, and 3.8 min, respectively.

#### Lesion quantification

In order to assess the impact of the halo-artifact on activity quantification, phantom measurements were performed with an additional prostate insert located in close vicinity to the bladder. The prostate insert allows adding several small spherical lesions with different activity uptake. For the experiment performed here, two 1.2 mL and 2.6 mL lesions were filled with activity. We used ^68^Ga for bladder (42 MBq), background (17 MBq) and lesions (activity concentrations compared to background: 100:1 and 10:1 for the 1.2 mL and the 2.6 mL lesion, respectively). Two measurements were performed with the mMR: with and without the bladder insert. With the bladder insert, PET data were acquired in list-mode (8 min acquisition time). After carefully removing the bladder without moving the phantom, a second PET scan was performed, acquiring 10^7^ counts after randoms correction (3.3 min data acquisition in sinogram mode).

### Patient data

The retrospective study was approved by the Institutional Review Board of Heidelberg (S-515/2016) in accordance with the Declaration of Helsinki and its later amendments. Written informed consent was waived. We investigated a total of 31 ^68^Ga-PSMA-11 patients (aged 65.0±6.9 years) who underwent PET/MRI of the abdomen 146±39 min p.i. (208±70 MBq). The patient inclusion criterion was either biochemical recurrence (PSA > 0.2 ng/ml) or presence of high-risk prostate carcinoma confirmed by biopsy. All patients were asked to void their bladder directly before PET raw data acquisition. PET raw data were acquired using 2–3 bed positions (4 min/bed position) covering upper to lower abdomen. Slice overlap between adjacent bed positions was 30 planes corresponding to 6.1 cm. The arms were positioned down, i.e., along the body. Vendor-provided standard MR-derived attenuation maps (two-point DIXON) were used as basis for attenuation and scatter correction.

### Data processing

#### Reconstruction

For reconstruction of the acquired PET raw data, the Siemens e7tools offline reconstruction package was used, versions VA20 (mMR) and VG40 (mCT), respectively. Reconstructions were performed employing the ordinary Poisson OSEM (OP-OSEM) algorithm [*30*] using 3 iterations and 21 subsets and accounting for randoms, scatter, normalization, and attenuation. For the phantom data acquired with the mCT, data were reconstructed twice: without and with TOF information. Transversal image matrix size of the reconstructed volumes was 344 × 344 (2.09 mm × 2.09 mm) for the mMR and 400 × 400 (2.04 mm × 2.04 mm) for the mCT. Slice thickness was 2.03 mm in both cases. All reconstructed volumes were smoothed employing 3D Gaussian post-smoothing with FWHM = 5 mm in all three dimensions.

#### Scatter estimation

The vendor-based implementation of the SSS algorithm was employed for scatter estimation. Default parameters were used and kept constant for all experiments (energy window: 430 to 610 keV, number of iterations: 4, additional scale factor: 1.0) with the exception of the parameters described in the following. We either used relative (Rel) or absolute (Abs) scaling of the estimated scatter, as described in the introduction section. In addition, we modified the maximum scatter fraction (MaxSF), which sets an artificial threshold to the estimated scatter fraction (SF), i.e., to the ratio of the SSS-based scatter estimate and the acquired prompt events. If the initially estimated SF after absolute or relative scaling exceeds MaxSF, then additional scaling of the entire scatter estimate is performed such that the final SF equals MaxSF. Therefore, from an algorithmic point of view, modifying MaxSF only changes this additional scatter downscaling. Employing a reduced value for MaxSF was motivated since SSS using a default MaxSF = 75%, as implemented by the vendor, tends to overestimate scatter in ^68^Ga-PSMA-PET, potentially resulting in halo-artifacts (Fig 1). In this work, we show results obtained with MaxSF = 40% and some with MaxSF = 30% along with the default value of MaxSf = 75%. Our choice MaxSF = 40% was motivated by previous work, publishing scatter fraction values of 30% to 60% based on phantom measurements [*27*,*31*–*33*], Monte Carlo simulations [*34*,*35*], and estimations on patient data [*19*,*36*–*38*]. The scatter fraction based on the NEMA NU 2–2007 protocol was found to be 37.9% for the Biograph mMR [*27*]. For the patient data, we employed absolute SSS as well as relative SSS with and without vendor-based PGC for ^68^Ga. All three variants were combined with either MaxSF = 75% or MaxSF = 40%, resulting in a total of six different scatter estimation methods and corresponding reconstructions (Abs75, Abs40, Rel75, Rel40, PGC75, PGC40).

#### Organ-to-background-ratio

To investigate the relation between OBR and halo size, the OBR was obtained empirically based on the reconstructed PET images employing the default scatter estimation (Abs75) and evaluating representative ROIs in either bladder or kidneys and the corresponding background.

#### Lesion quantification

For the experiment with bladder insert, only data corresponding to the first 7 min of data acquisition were used for reconstruction (data were acquired in list-mode). This was done such that the noise in the background was the same for the reconstruction with and without bladder. In addition, decay correction was performed to account for the time delay between the two consecutive scans. Different scatter estimation variants were applied (Abs75, Abs40, Abs30, Rel75, Rel40, Rel30) during reconstruction and their effect on lesion and background quantification was evaluated. We used manually segmented 3D ROIs representing the two lesions and the background. For the lesion in direct vicinity to the bladder, the ROI was chosen such as to avoid an overlap with the reconstructed bladder activity. For the background, the ROI was chosen such that it was not visually affected by halo-artifacts for either of the investigated scatter correction variants.

#### Quantitative evaluation

The halo was defined as volume of consecutive zero-valued voxels being located either around the kidneys or the bladder. For each reconstructed PET image, the halo was semi-automatically segmented with the image processing platform MITK [*39*], employing a 2D region-growing-algorithm in transversal, sagittal, and coronal slice orientation in combination with a 3D interpolation. The locations of the seed points were chosen manually to ensure that the majority of consecutive zero-valued voxels were segmented by the algorithm. Relevant segmentation parameters were kept constant for all PET data sets (upper/lower threshold = 0, multiplier = 0, neighborhood radius = 1). The OBR was then plotted against the halo size. For the phantom measurements, the data were fitted against a linear function.

#### Visual Evaluation

Based on the results of the quantitative evaluation, only the reconstructions corresponding to the scatter correction variants Rel75, Abs75, and Abs40 were evaluated by two independent readers with more than three years of experience in hybrid PET/MRI (M.T.F. and A.D.-S., the latter as expert reader with > 25 years of experience in PET imaging). The readers assigned scores according to the following scale: 0 (no artifact; not compromising image quality), 1 (small or faint artifact; tolerably compromising image quality), 2 (intermediate artifact; compromising image quality), 3 (large artifact; severely compromising image quality). Inter-reader agreement was determined calculating the average-measures intraclass correlation coefficient and its 95% confidence interval employing a two-way consistency-type model. 0.0–0.2 was defined as slight, 0.2–0.4 as moderate, 0.4–0.6 as fair, 0.6–0.8 as substantial, and 0.8–1.0 as near perfect agreement.

#### Statistical analysis

For each patient data set, halo sizes were normalized to the Rel75 reconstruction, which showed the largest halo-artifacts. Statistical analysis using MedCalc Software version 14 (MedCalc Software, Ostend, Belgium) was performed to evaluate which scatter correction variant (Abs75, Abs40, Rel40, PGC75, or PGC40) achieves the best halo-artifact suppression compared to Rel75. To analyze whether a given scatter correction variant was statistically significant compared to any other scatter correction variant, a Friedman-test including Conover post-hoc correction was performed for all patient data sets. A p-value less than 0.05 was considered statistically significant.

## Results

### Phantom measurements

#### PET/MRI vs. PET/CT and ^68^Ga vs. ^18^F-FDG

**Fig 2:**
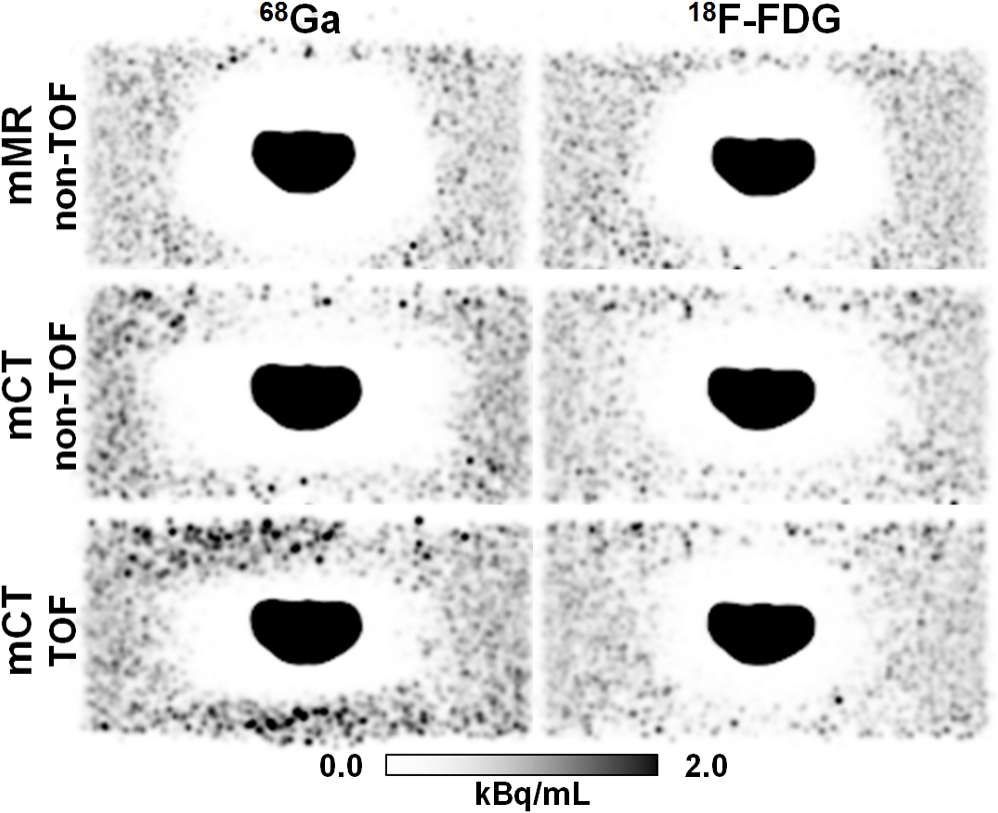
Comparison of halo-artifact appearance in PET/MR and PET/CT for ^68^Ga and ^18^F-FDG. PET images of the pelvis phantom in coronal slice orientation using absolute SSS with MaxSF = 75%. The administered tracer was either ^68^Ga or ^18^F-FDG and scans were performed subsequently in PET/CT (mCT) and PET/MRI (mMR). The mCT data were reconstructed twice: without and with TOF information. A severe halo-artifact surrounding the bladder insert was visible in all six cases.

The measured OBRs were around 700 in case of ^18^F-FDG and 850 in case of ^68^Ga. A qualitative evaluation of the reconstructed images corresponding to the default scatter estimation Abs75 showed a severe halo-artifact surrounding the bladder insert in all investigated cases (Fig 2). In the ^68^Ga-case, the halo seems to be slightly larger than for ^18^F-FDG, which may be attributed to the larger OBR. Apart from the slightly different OBR, there does not seem to be a dependency of the halo-artifact on the radioactive tracer which is applied. The observation that the halo appearance was very similar for both ^68^Ga and ^18^F-FDG also indicates that prompt gammas are not the primary reason for halo artifacts in ^68^Ga-PSMA-11-PET imaging. Although the shape of the observed artifacts slightly differs between PET/MR (mMR) and PET/CT (mCT), there does not seem to be a strong dependency on the measurement device. Incorporating TOF information into the reconstruction of the PET/CT data somewhat reduced the size of the halo-artifact.

#### Dual tracer experiments

**Fig 3:**
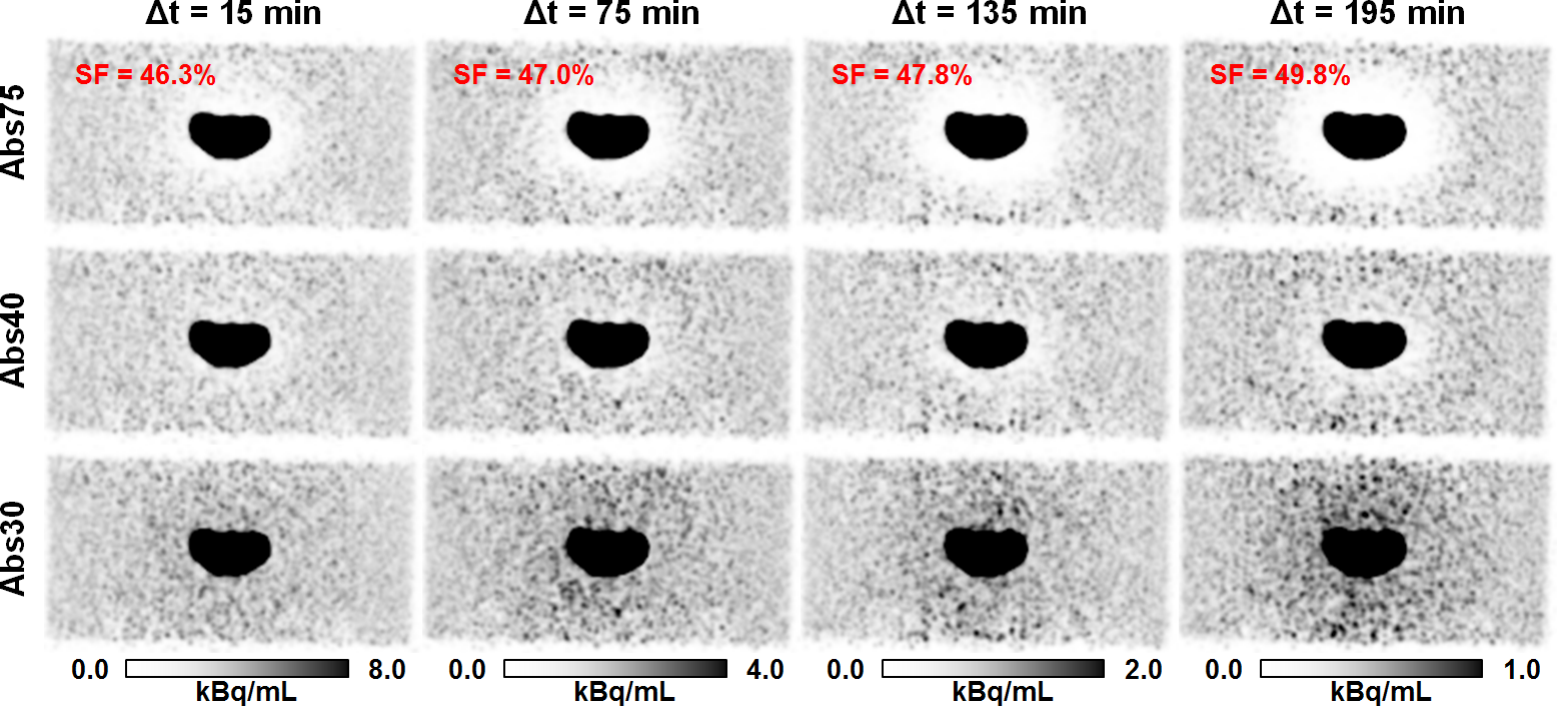
Effect of reduction of maximum scatter scatter fraction on halo-artifact appearance. PET images of the pelvis phantom in coronal slice orientation using absolute scatter scaling and both default (MaxSF = 75%) and reduced maximum scatter fraction (MaxSF = 40% or MaxSF = 30%). The administered tracer was ^68^Ga (33 MBq, background) and ^18^F-FDG (23 MBq, bladder insert). PET raw data were acquired with the mMR 15, 75, 135, and 195 min p.i., and corresponding OBRs were 108, 136, 184, and 248, respectively. The scatter fraction (SF) increased with increasing time, i.e., increasing OBR A halo-artifact increasing in size over time was observed when the default maximum scatter fraction (MaxSF = 75%) was applied. The halo-artifact was hardly visible for any measurement when using the reduced MaxSF = 40%. Further reduction of MaxSF, e.g., to 30% resulted in activity overestimation surrounding the bladder insert.

Results of the dual tracer experiment are shown in Fig 3. The initial measurement 15 min p.i. (OBR = 108) hardly showed any halo-artifact around the bladder insert. With increasing time, i.e., increasing OBR, the scatter fraction increased and so did the size of the halo-artifact when using the default MaxSF = 75%. In contrast, halo-artifacts were strongly suppressed for all acquired PET raw data sets corresponding to the different OBRs when using the reduced MaxSF = 40%. Reducing MaxSF to 30% resulted in a slight activity overestimation surrounding the bladder insert.

#### Lesion quantification

**Fig 4:**
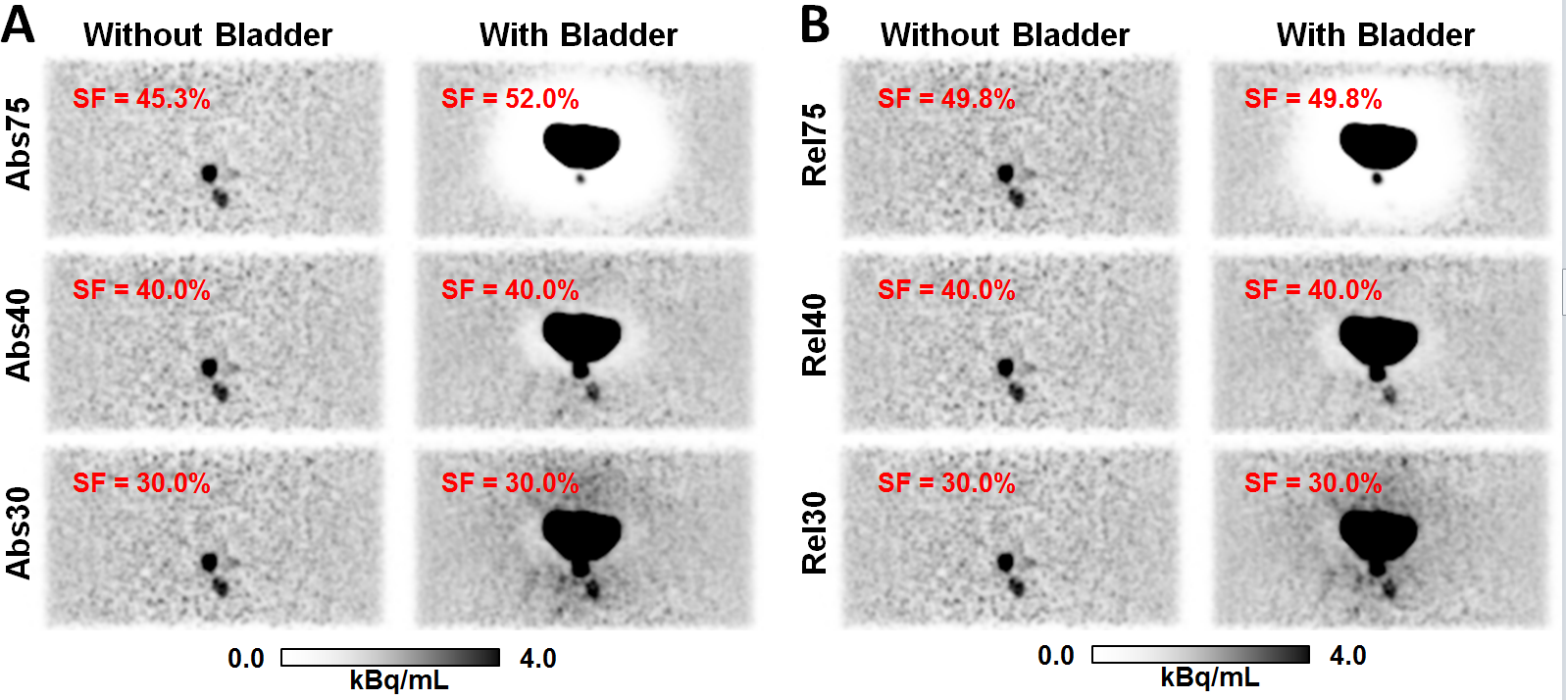
Effect of the halo-artifact on lesion quantification. PET images of the pelvis phantom in coronal slice orientation for the lesion quantification experiment using absolute (A) and relative (B) scatter scaling. Without the bladder insert, both lesions can be clearly identified. With bladder insert, a severe halo-artifact is observed using the default MaxSF = 75%, masking one of the two lesions. Quantitative numbers corresponding to the reconstructions shown are given in Table 1. SF specifies the scatter fraction for each case.

Results for the reconstruction based on different scatter estimation variants are presented in Fig 4. Without the bladder, no halo artifact was present. With bladder, a severe halo was found when using the default maximum scatter fraction MaxSF = 75%, preventing the detection of one of the lesions and significantly changing the quantification of the second lesion. Very similar results were observed for absolute and relative SSS. Table 1 states measured activity values evaluated in ROIs corresponding to the lesions and to the background. The reconstructions without bladder (i.e., without halo-artifact) and with MaxSF = 75% are considered as ground truth. Reducing MaxSF to 40% resulted in much improved lesion detectability and in improved activity quantification in the lesions. For example, lesion 2, which has an activity concentration of 10:1 compared to the background, was entirely missed with the default MaxSF = 75%. The relative mean activity uptake in the corresponding ROI was only 2.4% and 1.6% of the ground truth value for Abs and Rel, respectively. With MaxSF = 40% and 30%, the relative mean activity value could be increased to 85.7% and 115.4% for Abs and to 72.7% and 102.0% for Rel. The slightly better lesion quantification with MaxSF = 30% compared to MaxSF = 40% was counteracted by artificially increasing the background activity compared to the ground truth, which is also visible in the corresponding images in Fig 4.

**Table 1:** Maximum/Mean activity uptake values in kBq/mL evaluated in the two lesions and in the background for the reconstructions corresponding to the different scatter correction variants and without (w/o) and with (w) bladder insert.

Scatter correction variant	Lesion 1 (1.2 mL, 100:1)		Lesion 2 (2.6 mL, 10:1)		Background	
	w/o bladder	w bladder	w/o bladder	w bladder	w/o bladder	w bladder
Abs75	36.2/23.2	14.7/ 6.4	3.62/2.86	0.33/0.07	1.31/0.76	1.21/0.77
Abs40	36.4/23.3	29.4/21.4	3.84/3.06	3.67/2.45	1.43/0.82	1.46/0.86
Abs30	36.5/23.4	31.1/23.0	3.94/3.16	4.51/3.30	1.49/0.85	1.53/0.89
Rel75	36.5/23.4	19.9/10.2	3.85/3.07	0.24/0.05	1.26/0.77	1.47/0.83
Rel40	36.5/23.4	29.7/21.5	3.86/3.09	3.39/2.23	1.44/0.85	1.48/0.84
Rel30	36.6/23.5	31.3/23.2	3.92/3.14	4.29/3.13	1.51/0.88	1.51/0.86

#### OBR—Phantom evaluation

**Fig 5:**
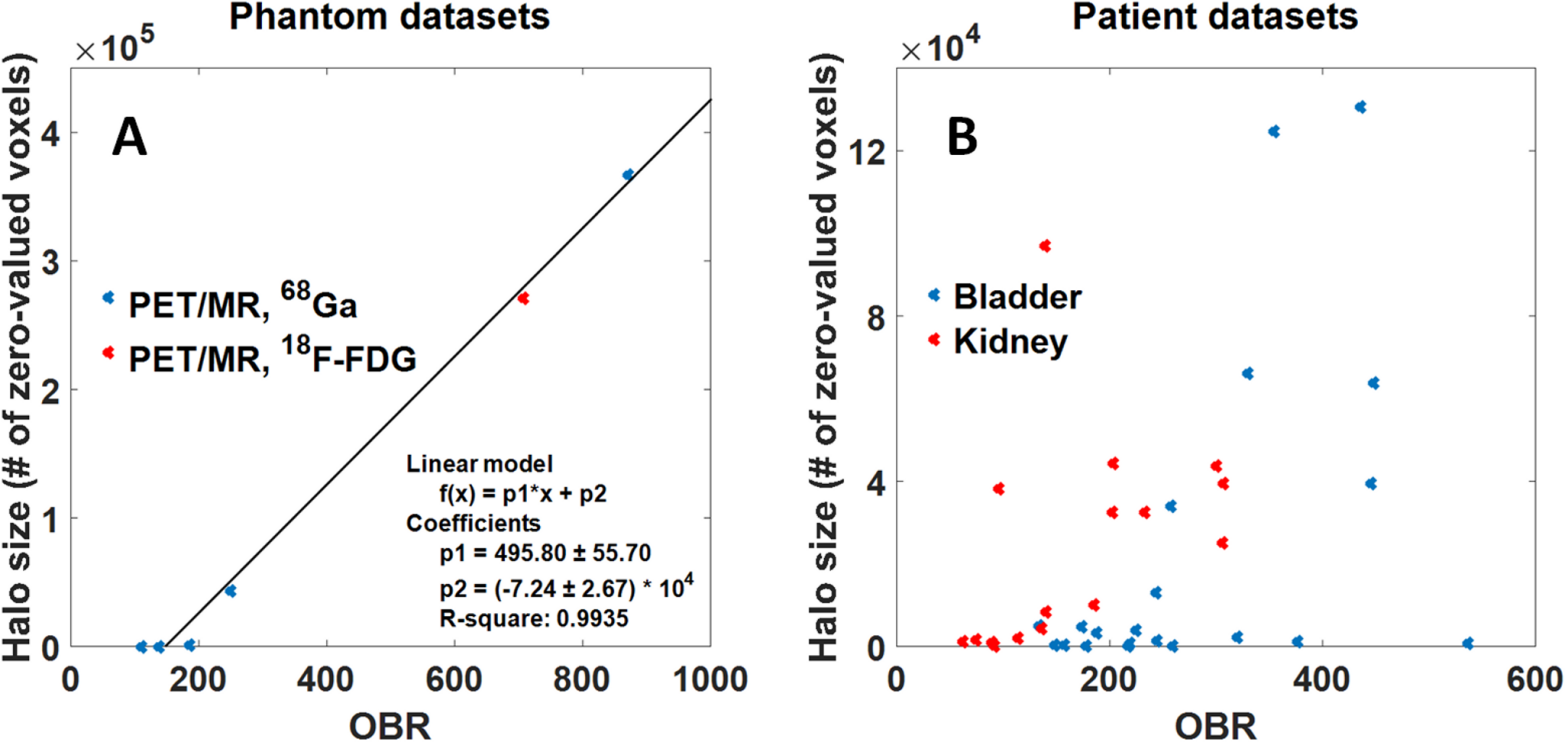
Relation between organ-to-background ratio (OBR) and halo size. (A) Correlation between OBR and halo size for different phantom experiments using either ^68^Ga (blue) or ^18^F-FDG (red) acquired on a PET/MRI system applying the default absolute SSS with MaxSF = 75%. OBR and halo size were fitted against a linear function. (B) Correlation between OBR and halo size for PET/MRI patient data sets for the Abs75 reconstruction. The halo size was calculated for a total number of 21 and 16 patients for bladder (blue) and kidneys (red), respectively. The data show a clear trend to larger halo sizes with increasing OBR.

Evaluating the entire set of phantom measurements, we found a linear relationship (R² = 0.9935) between OBR and halo size. A combined plot including PET/MRI data for both ^18^F-FDG and ^68^Ga and the corresponding coefficients of the linear fit are displayed in Fig 5A. No halo, defined as volume of consecutive zero-valued voxels, was observed for OBRs smaller than 150.

### Patient data

#### Quantitative evaluation

**Fig 6:**
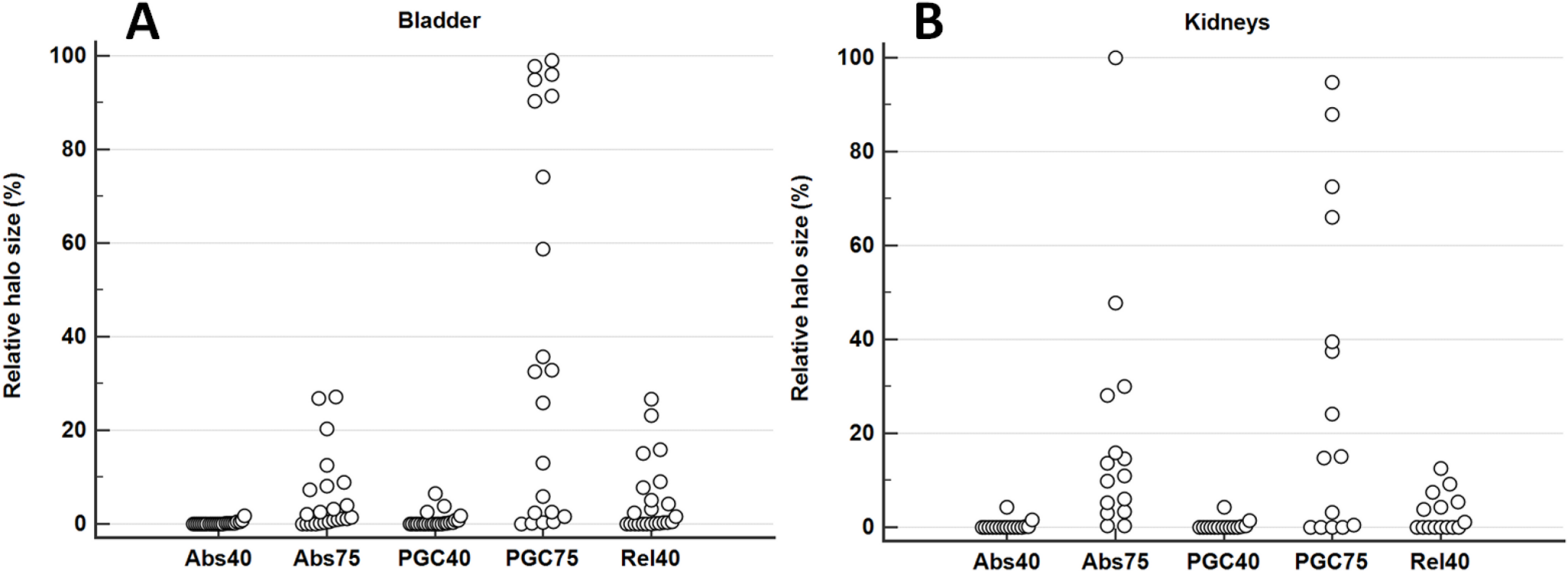
Evaluation of different scatter correction variants. For each patient data set, these graphs visualize the relative halo size for the different scatter correction variants compared to Rel75 (100%). A total of 21 patients were evaluated for the bladder (A) and 16 in case of the kidneys (B).

Measured OBRs ranged from 60 to 540 (bladder) and 40 to 310 (kidneys). Although a linear relation was not apparent in the patient data compared to the phantom data, a clear trend towards larger halo sizes was seen for higher OBRs (Fig 5B). Fig 6 shows the relative halo size for the different scatter correction variants in comparison to Rel75. For all variants of SSS (Abs, Rel, PGC), reducing MaxSF from 75% to 40% strongly reduced the size of the halo-artifact. The results of the statistical analysis are given in Table 2. For both the kidneys and the bladder, absolute SSS (Abs) and relative SSS combined with PGC are superior (statistically significant) to relative SSS (Rel), for both MaxSF = 75% and MaxSF = 40%. For the bladder, Abs proved to be significantly better than PGC while there was no statistical difference found between Abs and PGC in case of the kidneys. Moreover, reducing MaxSF from 75% to 40% significantly improved halo-artifact suppression for any scatter correction variant (Abs, Rel, PGC). Overall, Abs40 achieved the best halo-artifact suppression compared to any other scatter correction variant (Friedman rank, median).

**Table 2:** Statistical results for halo-artifact reduction of the different scatter correction variants.

(#) Scatter correction variant	Median [%]	Min … Max [%]	Friedman rank	Significantly different to other variants*
***Bladder (n = 21)***				
*(1)* Abs40	0.03	0 … 1.76	1.33	*(2–6)*
*(2)* PGC40	0.04	0 … 6.52	2.00	*(1*,*3–6)*
*(3)* Rel40	1.65	0 … 26.70	3.48	*(1*,*2*,*5*,*6)*
*(4)* Abs75	1.45	0 … 27.22	3.74	*(1*,*2*,*5*,*6)*
*(5)* PGC75	32.54	0 … 99.04	4.45	*(1–4*,*6)*
*(6)* Rel75	100	100	6.00	*(1–5)*
***Kidneys (n = 16)***				
*(1)* Abs40	0.00	0 … 4.29	1.88	*(3–6)*
*(2)* PGC40	0.00	0 … 4.34	2.06	*(3–6)*
*(3)* Rel40	0.04	0 … 12.62	2.75	*(1*,*2*,*4–6)*
*(4)* Abs75	10.34	0.1 … 100	4.38	*(1–3*,*6)*
*(5)* PGC75	14.94	0 … 94.76	4.00	*(1–3*,*6)*
*(6)* Rel75	100	100	5.94	*(1–5)*

Median, minimum and maximum values represent the ratio of zero-valued voxels in comparison to Rel75 (100%).

*Conover post-hoc correction (p<0.05) for Friedman’s analysis.

#### Visual evaluation

**Fig 7:**
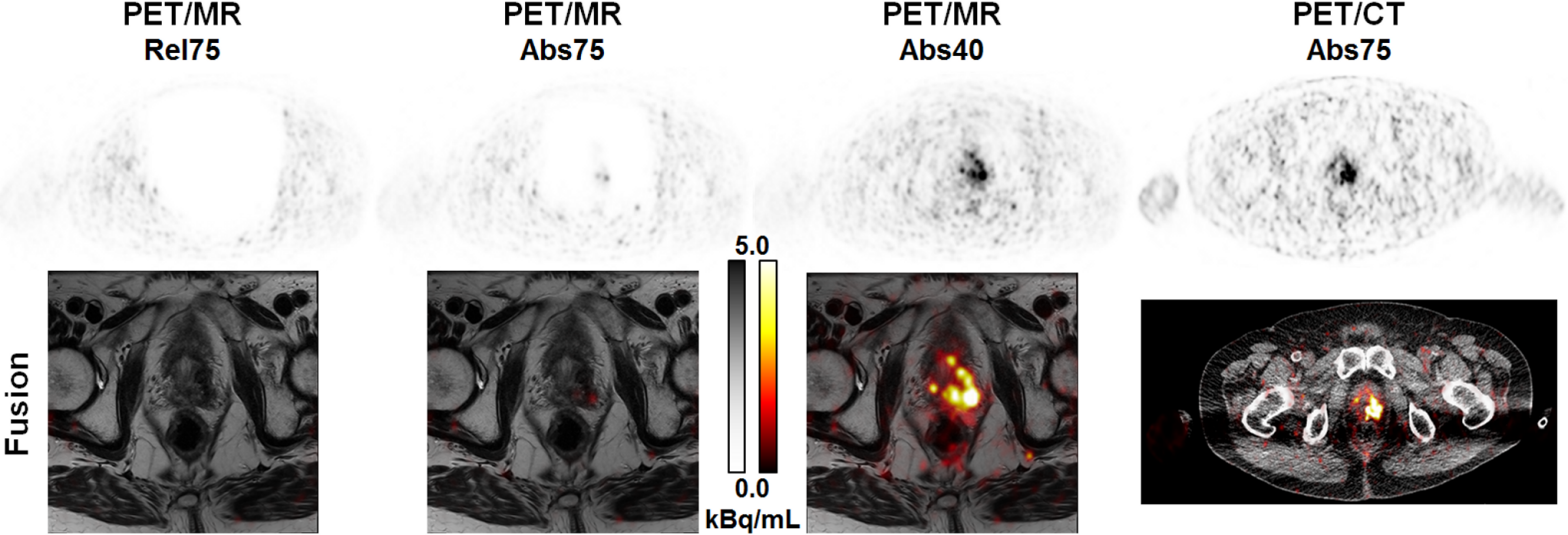
Reducing the maximum scatter fraction may change the patient’s diagnosis. 71 years old patient with biochemical recurrence of a prostate carcinoma (Gleason score 9, determined by biopsy) in the left lobe after primary radiation therapy (74 Gy). Administered activity was 189 MBq ^68^Ga-PSMA-11 and the patient was scanned with the mMR 179 min p.i. This example demonstrates the clinical impact of the halo-artifact resulting from high uptake ratio in the bladder and very low uptake in the abdominal soft tissue and fat (OBR = 330). In Rel75 and Abs75 reconstructions, the recurrence is not (Rel75) or only faintly (Abs75) detectable, both in the PET (top row) and the fused (bottom row) images. Reducing MaxSF to 40% is mandatory to avoid false-negative diagnosis in the PET component (the MRI component suggested recurrence). Reference is given by PET/CT 1h p.i. (OBR = 109).

**Fig 8:**
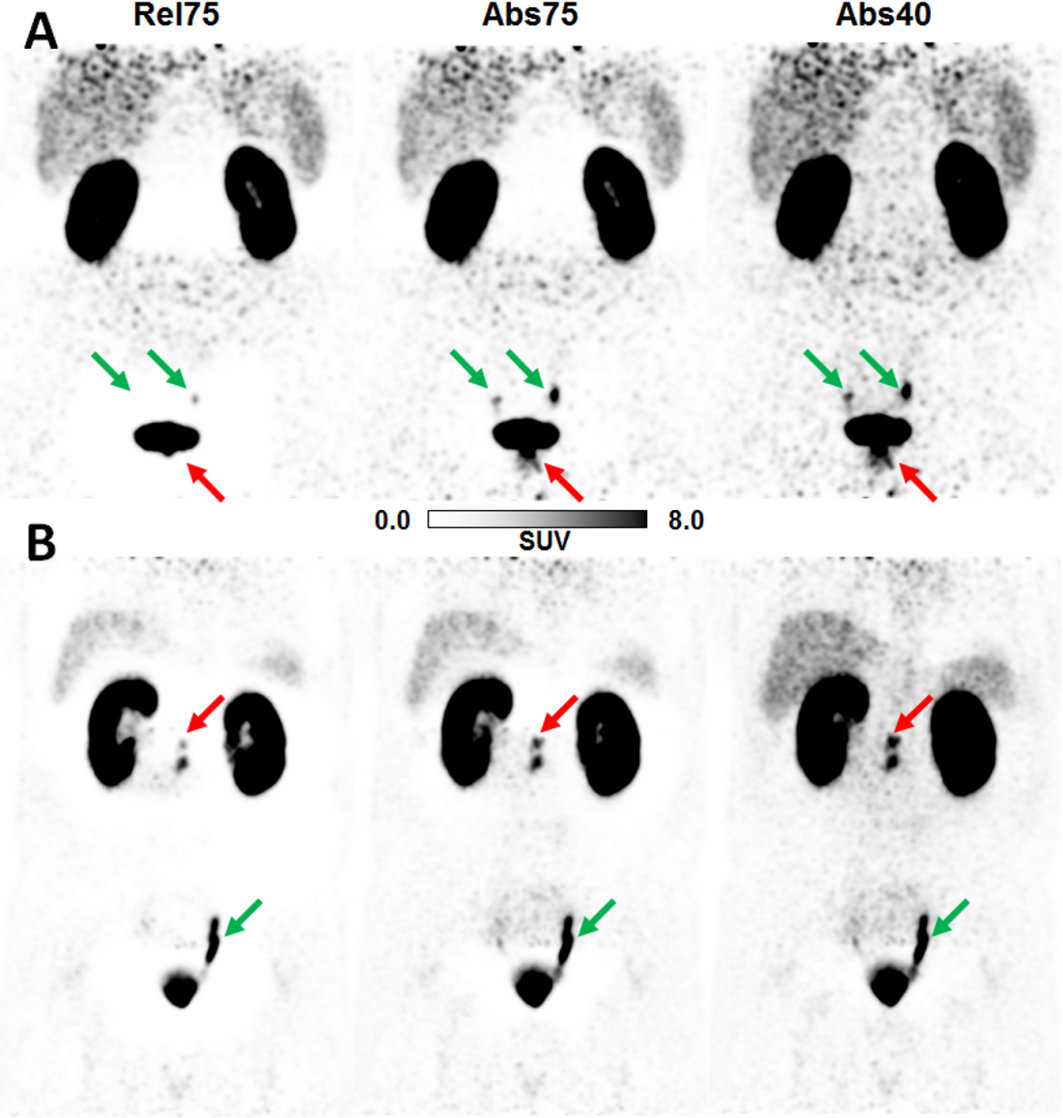
Effects of different scatter correction variants on PET quantification. (A) Patient (52 years, 160 MBq ^68^Ga-PSMA-11, 188 min p.i.) with prostate carcinoma (red arrow, Gleason score 9, confirmed by surgery). Mean and maximum standardized uptake values SUV_mean_/SUV_max_ values evaluated in the pathological lesion indicated by the red arrow: 0/0 (Rel75), 3.0/8.3 (Abs75), 4.3/10.6 (Abs40). (B) Patient (66 years, 237 MBq ^68^Ga-PSMA-11, 137 min p.i.) with biochemical recurrence after prostatectomy (red arrow, Gleason score 9, confirmed by surgery). SUV_mean_/SUV_max_ values evaluated in the pathological lesion indicated by the red arrow: 5.3/8.6 (Rel75), 9.1/14.1 (Abs75), 11.3/17.6 (Abs40). Green arrows indicate physiology (ureters). Standardized 3D-isocontour (40% of SUVmax) was used for SUV quantification.

The results of the visual evaluation are summarized in Table 3. Average scores assigned to the evaluated scatter correction variants across the two readers were 0.68±0.64 for Abs40, 2.18±0.96 for Abs75, and 2.65±0.77 for Rel75, revealing that absolute SSS is superior to relative SSS and reducing the maximum scatter fraction is highly beneficial. There was at least substantial inter-rater agreement for the three evaluated scatter correction variants. Agreement on Abs40 was slightly lower compared to the other two variants, probably because the readers disagreed on the impact of faint halos still present in some patient data sets when using the reduced MaxSF value. These results thus confirmed the findings obtained by the statistical analysis. The case of one specific patient, for whom the reduction of the maximum scatter fraction from 75% to 40% changed the diagnosis, is presented in Fig 7. Two more examples demonstrating the impact of different scatter correction variants on PET quantification are given in Fig 8, demonstrating the impact of the halo-artifact on lesions quantification.

**Table 3:** Results of the visual evaluation and inter-reader agreement.

Scatter correction variant	Score Reader 1	Score Reader 2	ICC* (95% CI)
Abs40	0.55±0.68	0.81±0.60	0.77 (0.47–0.88)
Abs75	2.00±1.03	2.35±0.88	0.87 (0.72–0.94)
Rel75	2.55±0.81	2.74±0.73	0.93 (0.85–0.96)

Results are given for Rel75 (default for, e.g., ^18^F-FDG), Abs75 (default for ^68^Ga-PSMA-11), and Abs40 (proposed for ^68^Ga-PSMA-11) (n = 31). The clinical impact of the halo-artifact was rated on a scale from 0 (no artifact) to 3 (large artifact).

*Intraclass correlation coefficient (ICC) and its 95% confidence interval (CI).

## Discussion

Despite ^68^Ga-PSMA-11-PET/MRI being an imaging method of high interest in oncological hybrid imaging of prostate cancer patients, its regular clinical use is impaired by photopenic artifacts surrounding organs of high uptake such as the bladder and the kidneys [*8*,*11*,*13*,*15*,*17*,*18*,*26*]. Evaluating our phantom measurements, we could clearly demonstrate that these so-called halo-artifacts are neither tracer-dependent (neglecting the physiological biodistribution) nor modality-dependent (neglecting TOF information and impact of improper attenuation maps). Since halo-artifacts were also present when ^18^F-FDG was used as tracer and since their size and appearance was comparable to the ^68^Ga-case, prompt gammas could be excluded as being the main reason for halo-artifacts. Nevertheless, prompt gamma correction (PGC) was found to be beneficial in some cases when combined with relative SSS (Fig 6, Table 2). However, PGC was sufficient to entirely remove the halo-artifact around the bladder in only 8 out of 21 patients and around the kidneys in only 6 out of 16 patients. For some patients, the effect of PGC was close to zero, especially for halo-artifacts around the bladder. Similar results have recently been published by Noto et al. [*18*]. Hong et al. observed significant improvements in halo-artifact appearance for ^68^Ga-PSMA-PET/CT when PGC was applied [*15*]. In their patient study, halo-artifacts around the kidneys could be entirely removed in 5 out 6 cases, with only a very faint halo still present for the sixth patient. The reason why PGC seems to be more effective in the PET/CT study as compared to the PET/MRI studies presented in this work as well as by Noto et al. [*18*] is most likely because of the different arm positioning (arms up for PET/CT and arms down for PET/MRI). In addition, patients were scanned up to 3 h p.i. in our study, potentially resulting in very high OBRs, especially around the bladder.

The phantom experiments also showed comparable halo-artifacts in PET/MRI and PET/CT. Since the impact of an inaccurate MRI-based attenuation map was excluded by using a PET/CT-derived attenuation map of the phantom, only the PET-components of the two modalities were compared. Incorporating TOF information into reconstruction of the PET/CT data resulted in slightly reduced halo-artifacts, indicating that TOF is able to partially compensate for data inconsistencies induced by inaccurate scatter estimation (Fig 2). This observation is in accordance with the literature, where benefits of TOF, apart from increased signal-to-noise ratios [*40*], have been reported in case of inconsistent data, e.g., for incorrect MR-based attenuation maps [*41*,*42*]. However, for the phantom experiments performed in this work, TOF information was not sufficient to entirely suppress halo-artifacts induced by incorrect scatter estimates. For patient data, inaccurate and truncated attenuation maps, the lack of TOF information and larger time differences between tracer injection and patient investigation, potentially resulting in increased OBRs, may be the reason why non-TOF PET/MRI is more prone to halo-artifacts than PET/CT with TOF capability.

The reason why halo-artifacts have been reported for ^68^Ga-PSMA-11 but not for ^18^F-FDG most likely lies in the completely different tracer accumulation in human tissue, with^68^Ga-PSMA-11 being much more specific, resulting in significantly higher OBRs in clinical practice. Both the phantom experiments and the patient data demonstrated a positive correlation between OBR and occurrence and size of the halo-artifact, i.e., increasing OBR results in increasing halo size. These observations suggest asking the patient to void his bladder directly before PET raw data acquisition, possibly under stimulation by diuretic medication [*8*]. Thereby, OBRs can, potentially, be minimized helping to suppress halo-artifacts, at least around the bladder.

**Fig 9:**
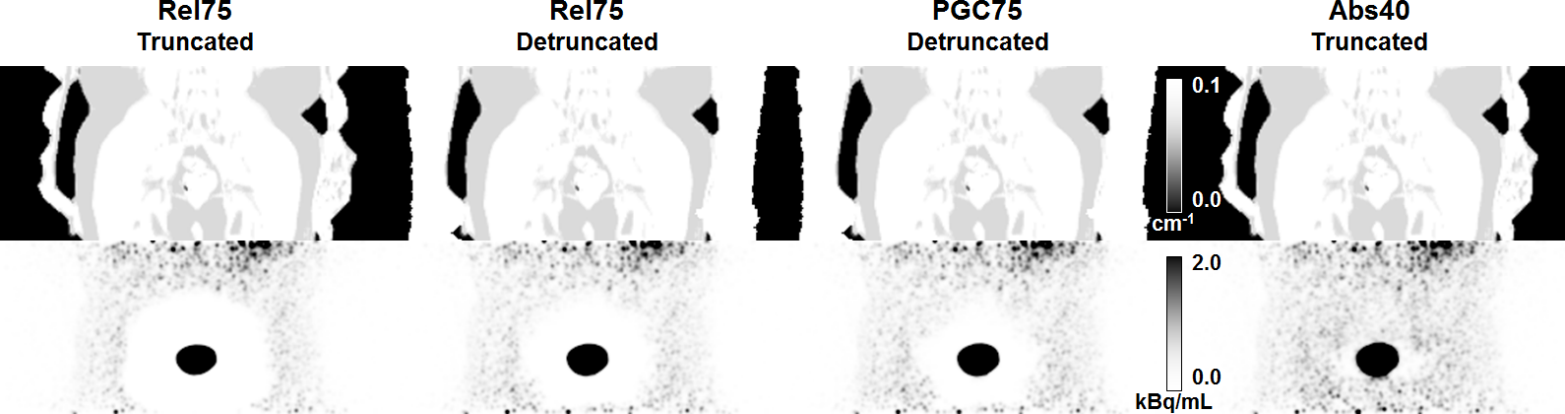
Effect of arm truncation on halo-artifact appearance. Attenuation maps and corresponding PET images of a clinical case acquired with a Biograph mMR in coronal slice orientation. In the standard MR-based attenuation maps, arms are truncated. Detruncation of the arms in the attenuation map significantly reduces the size of the halo-artifact for the applied relative (Rel) scaling. Additional prompt gamma correction (PGC) further reduces the halo-size but cannot completely compensate for the artifact, which potentially masks lesions in close vicinity to the bladder. For comparison, the reconstruction obtained with absolute scatter scaling and MaxSF = 40% is shown, which results in a complete halo-artifact suppression.

For the patient data, we used standard MR-derived attenuation maps, which neglect bone attenuation and often suffer from truncation, since the patients are usually scanned with their arms down as they were in our study. Two recent publications demonstrated that compensating for or avoiding arm truncation significantly improves the appearance of halo-artifacts in ^68^Ga-PSMA-PET/MRI [*18*,*26*]. However, both studies show that halo-artifacts may still be present, even when MLAA-based attenuation maps are employed [*18*] or when patients are scanned with their arms up [*26*]. We therefore expect that the results for the default MaxSF = 75% will improve significantly in terms of halo-artifact suppression if some truncation correction method, such as MLAA-based attenuation maps [*43*] or HUGE [*44*], is applied, especially in combination with relative scatter scaling (tail-fitting technique). Additional benefits with regard to halo-artifacts may be possible when using attenuation maps including bone information, e.g., derived from atlas-based methods [*45*]. However, the results of the phantom experiments suggest that inaccurate and especially truncated MR-based attenuation maps are not the only reason for halo-artifacts (an untruncated, CT-derived attenuation map was used for the phantom measurements). In addition, Fig 9 shows one example of a patient included in the present study, where detruncation of the attenuation map was not sufficient to entirely correct for halo-artifacts. For this example, detruncation was done manually by segmenting the arms in the non-attenuation-corrected PET images and assigning the corresponding voxels in the attenuation map with values corresponding to water.

Ruling out several potential causes as single or main reason for halo-artifacts in ^68^Ga-PSMA-11-PET/MRI (prompt gammas, truncated and inaccurate attenuation maps, non-TOF information) it remains open, what other causes for halo-artifacts are present. The implemented SSS algorithm does not explicitly model scatter events caused by activity outside the FOV. However, for the phantom experiments, the entire activity was within the FOV, both in transaxial and axial direction. This suggests that out-of-FOV activity is also to be ruled out as remaining main or single reason for halo-artifacts (although some effect cannot be excluded). Our phantom experiments did not provide any insight into the effect of multiple scatter. Including multiple scatter into the scatter estimation model would change both the shape and magnitude of the obtained scatter estimates and potentially improve halo-artifact appearance. Therefore, scatter correction algorithms explicitly treating multiple scatter should be investigated and, if proven valuable, should be made available in clinical practice.

For the phantom and patient data investigated in this study, both quantitative and visual evaluation showed that the default scatter correction variants, i.e., Rel75 (default for, e.g., ^18^F-FDG-PET/MRI) and Abs75 (default for ^68^Ga-PSMA-11-PET/MRI) do not sufficiently suppress halo-artifacts. Additionally, it was shown that reducing the maximum scatter fraction from MaxSF = 75% to MaxSF = 40% is highly beneficial to significantly reduce halo-artifacts, as visualized in Figs 3, 4, 7 and 8, and summarized in Tables 2 and 3. Further reduction of the maximum scatter fraction to 30% or lower resulted in a scatter underestimation for most data sets, apparent as locally increased activity values surrounding the bladder or the kidneys in the reconstructed PET images (Fig 3). Overall, both the quantitative and qualitative evaluation showed that a combination of the absolute variant of SSS with MaxSF = 40% (Abs40) resulted in the best halo-artifact suppression, both for the bladder and the kidneys. However, it should be considered that modifying MaxSF does not only affect the visual appearance and quantification in the region affected by halo-artifacts but throughout the entire image. As demonstrated by the lesion quantification experiment (Fig 4, Table 1), the effect on the background region, however, is significantly lower than on the halo-region. Nevertheless, it may be beneficial to consider images reconstructed with MaxSF = 75% alongside with those corresponding to MaxSF = 40% to ensure proper quantification both in the background as well as in regions affected by halo-artifacts.

As stated in the materials and methods section, reducing MaxSF changes the downscaling of the estimated scatter. Similarly, one could use an additional scaling factor *f* < 1.0 to reduce the magnitude of the estimated scatter (in fact, such a parameter is available with the Siemens e7tools; default value: 1.0). However, such a scaling would apply to all patients, regardless of the initial scatter fraction, whereas reducing MaxSF only changes the magnitude of the scatter if it is above the threshold specified by MaxSF. In other words, adjusting MaxSF is more flexible since it does not result in a downscaling of the scatter for patients with scatter fractions below MaxSF, and thus for cases which most likely do not suffer from halo-artifacts.

While the results presented in this work clearly demonstrate that reducing MaxSF improves image quality in terms of halo-artifacts and also improves quantification in the affected regions, the optimal choice of MaxSF is not so obvious. Our choice MaxSF = 40% was motivated by previously published studies estimating the scatter fraction based on phantom measurements [*27*,*31*–*33*], Monte Carlo simulations [*34*,*35*], and estimations on patient data [*19*,*36*–*38*]. The chosen value proved to work well for the phantom data and for all 31 patient data sets presented in this study. However, it should also be obvious that a single value for MaxSF may not reflect large differences in size across patients. For larger patients, higher values of MaxSF may be better suited to correct for halo-artifacts while at the same time minimizing the effect of the modified scatter scaling to other body regions. A similar idea was presented by Rezaei et al. at the last MIC conference, suggesting a plane-dependent scaling of the scatter estimate obtained by SSS, with the scaling parameters being chosen such that the likelihood function is maximized [*46*].

The clinical examples presented in Figs 7 and 8 demonstrate that halo-artifacts significantly influence the image appearance and, thus, the diagnosis. If the uptake of the pathological structure is very faint, it may be masked by halo-artifacts whereas high uptakes are usually detectable (Fig 7). Only in 1 out of 31 patients, a pathological lesion completely vanished in the default Abs75 reconstruction compared to the proposed Abs40. However, uptake values vary considerably, decrease with increasing size of the halo (Fig 8), and thus substantiate the necessity for correcting halo-artifacts to improve quantification in the affected regions. To avoid false-negative diagnoses caused by scatter-correction induced halo-artifacts, improved scatter correction methods producing halo-artifact-free PET images independent of the clinical workflow in ^68^Ga-PSMA-11-PET/MRI are mandatory. Our presented workaround based on a reduced maximum scatter fraction does not entirely solve the problem of halo-artifacts because it does not target the underlying problem of inaccurate scatter estimation. However, it significantly improves the image quality in terms of halo-artifact suppression and thus helps to improve scientific and clinical evaluation. Images reconstructed with a reduced maximum scatter fraction, e.g., MaxSF = 40%, should be made available in clinical practice and considered in cases were halo-artifacts are present, potentially alongside with images corresponding to the default MaxSF = 75%.

## Conclusion

^68^Ga-PSMA-11-PET/MRI is prone to halo-artifacts surrounding the bladder and the kidneys, which are caused by improper scatter correction and appear with increasing OBR over time. Because their appearance may be detrimental to image interpretation and thus patient diagnosis, strategies to avoid halo-artifacts are recommended. Besides striving for low OBRs we recommend reducing the maximum scatter fraction from 75% to 40% for the currently implemented SSS algorithm, which was shown to efficiently suppress halo-artifacts in all investigated cases. This parameter modification may serve as ad-hoc method to enable clinical ^68^Ga-PSMA-11-PET/MRI in patients where halo-artifacts are present until a more accurate scatter correction technique becomes available in clinical practice.

## References

[1] Silver DA, Pellicer I, Fair WR, Heston WWD, Cordon-Cardo C. Prostate-specific membrane antigen expression in normal and malignant human tissues. Clin Cancer Res. 1997;3:81–85. 9815541

[2] Sweat SD, Pacelli A, Murphy GP, Bostwick DG. Prostate-specific membrane antigen expression is greatest in prostate adenocarcinoma and lymph node metastases. Urology. 1998;52:637–640. 9763084 10.1016/s0090-4295(98)00278-7

[3] Eder M, Eisenhut M, Babich J, Haberkorn U. PSMA as a target for radiolabelled small molecules. Eur J Nucl Med Mol Imaging. 2013;40:819–823. doi: 10.1007/s00259-013-2374-2 23463331 10.1007/s00259-013-2374-2 PMC3644196

[4] Eder M, Schäfer M, Bauder-Wüst U, Hull W-E, Wängler C, Mier W, et al 68Ga-complex lipophilicity and the targeting property of a urea-based PSMA inhibitor for PET imaging. Bioconjug Chem. 2012;23:688–697. doi: 10.1021/bc200279b 22369515 10.1021/bc200279b

[5] Afshar-Oromieh A, Malcher A, Eder M, Eisenhut M, Linhart HG, Hadaschik BA, et al PET imaging with a [68Ga]gallium-labelled PSMA ligand for the diagnosis of prostate cancer: biodistribution in humans and first evaluation of tumour lesions. Eur J Nucl Med Mol Imaging. 2013;40:486–495. doi: 10.1007/s00259-012-2298-2 23179945 10.1007/s00259-012-2298-2

[6] Afshar-Oromieh A, Hetzheim H, Kratochwil C, Benesova M, Eder M, Neels OC, et al The novel theranostic PSMA-ligand PSMA-617 in the diagnosis of prostate cancer by PET/CT: biodistribution in humans, radiation dosimetry and first evaluation of tumor lesions. J Nucl Med. 2015;56:1697–1705. doi: 10.2967/jnumed.115.161299 26294298 10.2967/jnumed.115.161299

[7] Eiber M, Maurer T, Souvatzoglou M, Beer a. J, Ruffani A, Haller B, et al Evaluation of hybrid 68Ga-PSMA-ligand PET/CT in 248 patients with biochemical recurrence after radical prostatectomy. J Nucl Med. 2015;56:668–674. doi: 10.2967/jnumed.115.154153 25791990 10.2967/jnumed.115.154153

[8] Rauscher I, Maurer T, Fendler WP, Sommer WH, Schwaiger M, Eiber M. 68Ga-PSMA ligand PET/CT in patients with prostate cancer: How we review and report. Cancer Imaging. 2016;16:14 doi: 10.1186/s40644-016-0072-6 27277843 10.1186/s40644-016-0072-6 PMC4898465

[9] Afshar-Oromieh A, Avtzi E, Giesel FL, Holland-Letz T, Linhart HG, Eder M, et al The diagnostic value of PET/CT imaging with the 68Ga-labelled PSMA ligand HBED-CC in the diagnosis of recurrent prostate cancer. Eur J Nucl Med Mol Imaging. 2014;42:197–209. doi: 10.1007/s00259-014-2949-6 25411132 10.1007/s00259-014-2949-6 PMC4315487

[10] Schwenck J, Rempp H, Reischl G, Kruck S, Stenzl A, Nikolaou K, et al Comparison of 68Ga-labelled PSMA-11 and 11C-choline in the detection of prostate cancer metastases by PET/CT. Eur J Nucl Med Mol Imaging. 2017;44:92–101. doi: 10.1007/s00259-016-3490-6 27557844 10.1007/s00259-016-3490-6

[11] Afshar-Oromieh A, Haberkorn U, Schlemmer HP, Fenchel M, Eder M, Eisenhut M, et al Comparison of PET/CT and PET/MRI hybrid systems using a 68Ga-labelled PSMA ligand for the diagnosis of recurrent prostate cancer: initial experience. Eur J Nucl Med Mol Imaging. 2014;41:887–897. doi: 10.1007/s00259-013-2660-z 24352789 10.1007/s00259-013-2660-z

[12] Eiber M, Nekolla SG, Maurer T, Weirich G, Wester H-J, Schwaiger M. (68)Ga-PSMA PET/MR with multimodality image analysis for primary prostate cancer. Abdom Imaging. 2014;40:897–898. 10.1007/s00261-014-0301-z 25412869

[13] Freitag MT, Radtke JP, Hadaschik BA, Kopp-Schneider A, Eder M, Kopka K, et al Comparison of hybrid 68Ga-PSMA PET/MRI and 68Ga-PSMA PET/CT in the evaluation of lymph node and bone metastases of prostate cancer. Eur J Nucl Med Mol Imaging. 2016;43:70–83. doi: 10.1007/s00259-015-3206-3 26508290 10.1007/s00259-015-3206-3

[14] Minamimoto R, Hancock S, Schneider B, Chin F, Jamali M, Loening AM, et al Pilot Comparison of 68Ga-RM2 PET and 68Ga-PSMA PET in Patients with Biochemically Recurrent Prostate Cancer. J Nucl Med. 2015:557–563. doi: 10.2967/jnumed.115.168393 26659347 10.2967/jnumed.115.168393

[15] Hong I, Rothfuss H, Michel C, Casey M. Prompt Gamma Correction on Ga-68 PSMA PET Studies. In: IEEE Medical Imaging Conference Record.; 2015.

[16] Derlin T, Weiberg D, von Klot C, Wester HJ, Henkenberens C, Ross TL, et al 68Ga-PSMA I&T PET/CT for assessment of prostate cancer: evaluation of image quality after forced diuresis and delayed imaging. Eur Radiol. 2016;26:4345–4353. doi: 10.1007/s00330-016-4308-4 27011373 10.1007/s00330-016-4308-4

[17] Lütje S, Blex S, Gomez B, Schaarschmidt BM, Umutlu L, Forsting M, et al Optimization of acquisition time of 68Ga-PSMA-ligand PET/MRI in patients with local and metastatic prostate cancer. PLoS One. 2016;11:e0164392 doi: 10.1371/journal.pone.0164392 27755548 10.1371/journal.pone.0164392 PMC5068705

[18] Noto B, Büther F, Auf der Springe K, Avramovic N, Heindel W, Schäfers M, et al Impact of PET acquisition durations on image quality and lesion detectability in whole-body 68Ga-PSMA PET-MRI. EJNMMI Res. 2017;7:12 doi: 10.1186/s13550-017-0261-8 28168589 10.1186/s13550-017-0261-8 PMC5293699

[19] Watson CC, Newport D, Casey ME, Dekemp RA, Beanlands RS, Schmand M. Evaluation of simulation-based scatter correction for 3-D PET cardiac imaging. IEEE Trans Nucl Sci. 1997;44:90–97.

[20] Watson CC. New, faster, image-based scatter correction for 3-D PET. IEEE Trans Nucl Sci. 2000;47:1587–1594.

[21] Lederer CM, Shirley VS, Browne E. Table of Isotopes. 7th ed. New York: Wiley; 1978.

[22] Martin CC, Christian BT, Satter MR, Nickerson LDH, Nickles RJ. Quantitative PET with positron emitters that emit prompt gamma rays. IEEE Trans Med Imaging. 1995;14:681–687. doi: 10.1109/42.476109 18215872 10.1109/42.476109

[23] Walrand S, Jamar F, Mathieu I, Camps J, Lonneux M, Sibomana M, et al Quantitation in PET using isotopes emitting prompt single gammas: application to yttrium-86. Eur J Nucl Med Mol Imaging. 2003;30:354–361. doi: 10.1007/s00259-002-1068-y 12634962 10.1007/s00259-002-1068-y

[24] Beattie BJ, Finn RD, Rowland DJ, Pentlow KS. Quantitative imaging of bromine-76 and yttrium-86 with PET: a method for the removal of spurious activity introduced by cascade gamma rays. Med Phys. 2003;30:2410–2423. doi: 10.1118/1.1595599 14528963 10.1118/1.1595599

[25] Watson C, Hayden C, Casey M, Hamill J, Bendriem B. Prompt gamma correction for improved quantification in 82Rb PET. J Nucl Med. 2008;49:64P.

[26] Afshar-Oromieh A, Wolf M, Haberkorn U, Kachelrie? M, Gnirs R, Kopka K, et al Effects of arm truncation on the appearance of the halo artifact in 68Ga-PSMA-11 (HBED-CC) PET/MRI. Eur J Nucl Med Mol Imaging. 2017;11. 10.1007/s00259-017-3718-0 28508120

[27] Delso G, Fürst S, Jakoby B, Ladebeck R, Ganter C, Nekolla SG, et al Performance measurements of the Siemens mMR integrated whole-body PET/MR scanner. J Nucl Med. 2011;52:1914–1922. doi: 10.2967/jnumed.111.092726 22080447 10.2967/jnumed.111.092726

[28] Jakoby BW, Bercier Y, Conti M, Casey ME, Bendriem B, Townsend DW. Physical and clinical performance of the mCT time-of-flight PET/CT scanner. Phys Med Biol. 2011;56:2375–2389. doi: 10.1088/0031-9155/56/8/004 21427485 10.1088/0031-9155/56/8/004

[29] Mann P, Heußer T, de las Heras Gala H, Kachelrieß M, Bachert P. A hybrid imaging phantom for research applications and quality control for PET/MR and PET/CT systems. In: ESMRMB 2015, 32nd Annual Scientific Meeting. ESMRMB; 2015:S139.

[30] Comtat C, Bataille F, Michel C, Jones JP, Sibomana M, Janeiro L, et al. OSEM-3D Reconstruction strategies for the ECAT HRRT. In: IEEE Nuclear Science Symposium and Medical Imaging Conference (NSS/MIC).; 2004:3492–3496.

[31] Lartizien C, Comtat C, Kinahan PE, Ferreira N, Bendriem B, Trébossen R. Optimization of injected dose based on noise equivalent count rates for 2- and 3-dimensional whole-body PET. J Nucl Med. 2002;43:1268–1278. 12215569

[32] Ferrero A, Poon JK, Chaudhari AJ, MacDonald LR, Badawi RD. Effect of object size on scatter fraction estimation methods for PET-A computer simulation study. IEEE Trans Nucl Sci. 2011;58:82–86.

[33] Poon JK, Dahlbom ML, Moses WW, Balakrishnan K, Wang W, Cherry SR, et al Optimal whole-body PET scanner configurations for different volumes of LSO scintillator: a simulation study. Phys Med Biol. 2012;57:4077–4094. doi: 10.1088/0031-9155/57/13/4077 22678106 10.1088/0031-9155/57/13/4077 PMC3786676

[34] Adam L-E, Karp JS, Brix G. Investigation of scattered radiation in 3D whole-body positron emission tomography using Monte Carlo simulations. Phys Med Biol. 1999;44:2879–2895. 10616142 10.1088/0031-9155/44/12/302

[35] Hosokawa S, Inoue K, Kano D, Shimizu F, Koyama K, Nakagami Y, et al A simulation study for estimating scatter fraction in whole-body 18F-FDG PET/CT. Radiol Phys Technol. 2016:1–9. 28032297 10.1007/s12194-016-0386-x

[36] Watson CC, Casey ME, Michel C, Bendriem B. Advances in scatter correction for 3D PET/CT. In: IEEE Nuclear Science Symposium Conference Record.; 2004:3008–3012.

[37] Accorsi R, Adam L, Werner ME, Karp JS. Implementation of a Single Scatter Simulation Algorithm for 3D PET : Application to Emission and Transmission Scanning. IEEE Nucl Sci Symp Conf Rec. 2002;2:816–820.

[38] Accorsi R, Adam L-E, Werner ME, Karp JS. Optimization of a fully 3D single scatter simulation algorithm for 3D PET. Phys Med Biol. 2004;49:2577–2598. 15272675 10.1088/0031-9155/49/12/008

[39] Nolden M, Zelzer S, Seitel A, Wald D, Müller M, Franz AM, et al The Medical Imaging Interaction Toolkit: challenges and advances. Int J Comput Assist Radiol Surg. 2013;8:607–620. doi: 10.1007/s11548-013-0840-8 23588509 10.1007/s11548-013-0840-8

[40] Aklan B, Jakoby BW, Watson CC, Braun H, Ritt P, Quick HH. GATE Monte Carlo simulations for variations of an integrated PET/MR hybrid imaging system based on the Biograph mMR model. Phys Med Biol. 2015;60:4731–4752. doi: 10.1088/0031-9155/60/12/4731 26040657 10.1088/0031-9155/60/12/4731

[41] Conti M. Why is TOF PET reconstruction a more robust method in the presence of inconsistent data? Phys Med Biol. 2011;56:155–168. doi: 10.1088/0031-9155/56/1/010 21119224 10.1088/0031-9155/56/1/010

[42] Mehranian A, Zaidi H. Impact of time-of-flight PET on quantification errors in MR imaging-based attenuation correction. J Nucl Med. 2015;56:635–641. doi: 10.2967/jnumed.114.148817 25745090 10.2967/jnumed.114.148817

[43] Nuyts J, Bal G, Kehren F, Fenchel M, Michel C, Watson C. Completion of a truncated attenuation image from the attenuated PET emission data. IEEE Trans Med Imaging. 2013;32:237–246. doi: 10.1109/TMI.2012.2220376 23014717 10.1109/TMI.2012.2220376

[44] Blumhagen JO, Braun H, Ladebeck R, Fenchel M, Faul D, Scheffler K, et al Field of view extension and truncation correction for MR-based human attenuation correction in simultaneous MR/PET imaging. Med Phys. 2014;41:22303. 10.1118/1.4861097 24506641

[45] Paulus DH, Quick HH, Geppert C, Fenchel M, Zhan Y, Hermosillo G, et al Whole-body PET/MR imaging: quantitative evaluation of a novel model-based MR attenuation correction method including bone. J Nucl Med. 2015;56:1061–1066. doi: 10.2967/jnumed.115.156000 26025957 10.2967/jnumed.115.156000 PMC4894503

[46] Rezaei A, Salvo K, Panin V, Koesters T, Casey M, Boada F, et al. Plane-dependent ML scatter scaling: 3D extension of the 2D simulated single scatter estimate. In: IEEE Medical Imaging Conference Record.; 2016:M07-6.

